# Adolescent Peer Influence on Eating Behaviors via Social Media: Scoping Review

**DOI:** 10.2196/19697

**Published:** 2021-06-03

**Authors:** Alicia Chung, Dorice Vieira, Tiffany Donley, Nicholas Tan, Girardin Jean-Louis, Kathleen Kiely Gouley, Azizi Seixas

**Affiliations:** 1 Center for Early Childhood Health and Development Department of Population Health NYU Grossman School of Medicine New York, NY United States; 2 NYU Grossman School of Medicine New York, NY United States; 3 SUNY Downstate College of Medicine Brooklyn, NY United States

**Keywords:** social media, eating behaviors, adolescent health

## Abstract

**Background:**

The influence of social media among adolescent peer groups can be a powerful change agent.

**Objective:**

Our scoping review aimed to elucidate the ways in which social media use among adolescent peers influences eating behaviors.

**Methods:**

A scoping review of the literature of articles published from journal inception to 2019 was performed by searching PubMed (ie, MEDLINE), Embase, CINAHL, PsycINFO, Web of Science, and other databases. The review was conducted in three steps: (1) identification of the research question and clarification of criteria using the population, intervention, comparison, and outcome (PICO) framework; (2) selection of articles from the literature using the PRISMA (Preferred Reporting Items for Systematic Reviews and Meta-Analyses) guidelines; and (3) charting and summarizing information from selected articles. PubMed’s Medical Subject Headings (MeSH) and Embase’s Emtree subject headings were reviewed along with specific keywords to construct a comprehensive search strategy. Subject headings and keywords were based on adolescent age groups, social media platforms, and eating behaviors. After screening 1387 peer-reviewed articles, 37 articles were assessed for eligibility. Participant age, gender, study location, social media channels utilized, user volume, and content themes related to findings were extracted from the articles.

**Results:**

Six articles met the final inclusion criteria. A final sample size of 1225 adolescents (aged 10 to 19 years) from the United States, the United Kingdom, Sweden, Norway, Denmark, Portugal, Brazil, and Australia were included in controlled and qualitative studies. Instagram and Facebook were among the most popular social media platforms that influenced healthful eating behaviors (ie, fruit and vegetable intake) as well as unhealthful eating behaviors related to fast food advertising. Online forums served as accessible channels for eating disorder relapse prevention among youth. Social media influence converged around four central themes: (1) visual appeal, (2) content dissemination, (3) socialized digital connections, and (4) adolescent marketer influencers.

**Conclusions:**

Adolescent peer influence in social media environments spans the spectrum of healthy eating (ie, pathological) to eating disorders (ie, nonpathological). Strategic network-driven approaches should be considered for engaging adolescents in the promotion of positive dietary behaviors.

## Introduction

### Defining Social Media

Adolescent peer groups have been recognized to influence individuals’ health behaviors, including diet [1]. During adolescence, eating behaviors are influenced by peer impacts, such as perceived social norms that can create unique peer pressures [2,3]. Peer-to-peer influence on health behaviors has been documented in face-to-face interactions [4]; however, few have studied the influence of social media on eating behaviors during adolescence.

Social media has been defined as any social networking site that enables interactive, user-generated content that allows sharing of images, ideas, videos, music, or commentary on internet forums (eg, Facebook), blogs and microblogs (eg, Twitter), and photograph- or video-hosting platforms (eg, Instagram, YouTube, or TikTok) [5]. Individuals or groups of people can communicate, collaborate, and connect in real time via text, video, or phone anywhere that Wi-Fi is available. Social media channels, such as Facebook or YouTube, were initiated in the early 2000s. However, the first website recognized as being the first social media platform was called *Six Degrees*—short for Six Degrees of Separation—and it launched in 1997. In 2018, YouTube, Instagram, and Snapchat were identified as the most popular online platforms utilized by teens 13 to 17 years of age [6]. User-generated content on these channels may allow for autonomy, identity, and interpersonal peer relationship development, a hallmark of adolescence [7].

Social media is an effective channel for engaging adolescents [8], a target population that has been hard to engage in public health practice. It can be used to influence, inform, and persuade. Social media mobile apps have global reach, use, and engagement [9]. In an earlier global report, approximately 85% of adolescents between the ages of 12 and 17 years across Europe, Latin America, the United States, and South Korea reported using a social media website [10]. Among a sample of 4460 high school students from Turkey in 2019, 88% owned a smartphone and 100% had a social media account [11]. Contagion effect—the rapid communication of an idea that has gone *viral* among peers on social media platforms—has been recognized as an effective way to promote health behaviors [12-15]. Behavior intent, increased knowledge, and increased awareness are positive attributes of healthful food posts on social media that influence users [16-18]. Extensive social media use, along with other entertainment media use, has been associated with consumption of unhealthy foods, mostly due to snacking behaviors. In particular, Albert found that social media and other entertainment media use among a sample of mostly Latino (68%) middle schoolers was negatively correlated with fruit and vegetable consumption (*r*=–0.065) and was strongly correlated with fast food and junk food intake (*r*<0.200) [19]. In a recent report, Chau et al concluded that social media was a promising channel for obesity prevention in adolescents and young adults [20]. Given that more recent research revealed that 95% of teens 13 to 17 years of age own a smartphone, 51% use Facebook, 69% use Snapchat, 72% use Instagram, and 85% use YouTube [21], an examination of peer influence, via social media channels, on eating behaviors is warranted. However, no review to date has demonstrated peer influence on eating behaviors via social media networks among adolescents.

### Social Media Influence and Eating Behaviors

A social network analysis of adult, in-person peer relationship influences indicated that maladaptive eating behaviors (ie, eating disorders) may be influenced by friendships [22]. Social norms, as well as real and perceived social support, may be underpinning peer influences related to the practice of eating. Peer groups and the type and degree of peer influence may shape one’s relationship with food. Peer influence on eating behaviors may extend from in-person influence to social media influence. Findings from a US nationally representative sample of young adults, 19 to 32 years of age, revealed an association between a high volume and frequency of social media platform engagement (ie, Facebook, Twitter, Google+, YouTube, LinkedIn, Instagram, Pinterest, Tumblr, Vine, Snapchat, and Reddit) and eating concerns [23]. However, some of the most popular social media channels have been noted to influence maladaptive (ie, nonpathological) eating disorders as well as adaptive (ie, pathological) healthy eating.

Social media platforms (ie, Facebook and YouTube) and mobile gaming nutrition-intervention apps (eg, Food Hero) demonstrate utility among young adult populations to raise awareness, increase knowledge, influence intrinsic beliefs, and motivate attitudes [23]. Social media channels, including Facebook, YouTube, and Snapchat, have been recognized by adolescents for providing peer-to-peer support in healthy eating through sharing information and offering social support [24]. This scoping review aimed to elucidate the role of peer influence via social media channels on eating behaviors among adolescents between the ages of 10 and 19 years.

## Methods

### Databases

The following databases were searched in October 2017 and updated in October 2019: PubMed (ie, MEDLINE), AgeLine, BIOSIS Citation Index, CINAHL, the Cochrane Library, Embase, ERIC (Education Resources Information Center), Food Science and Technology Abstracts, Google Scholar, Inspec, PubMed Central, PsycINFO, SciELO (Scientific Electronic Library Online), and Web of Science. PubMed’s Medical Subject Headings (MeSH) and Embase’s Emtree subject headings were reviewed along with specific keywords to construct a comprehensive search strategy. Grey literature was searched for in The New York Academy of Medicine Grey Literature Report and the OAIster database from the OCLC (Online Computer Library Center). An extensive electronic journal hand search was conducted in the following journals: American Journal of Health Promotion, American Journal of Preventive Medicine, Appetite, Childhood Obesity, Eating Behavior, Ethnicity & Disease, Ethnicity & Health, International Journal of Eating Disorders, International Journal of Obesity (London), Journal of the American Dietetic Association, Pediatrics, Obesity (Silver Spring), and Public Health Nutrition. In consultation with the first author (AC), a clinical librarian (DV) trained in systematic literature reviews conducted the literature search and managed the information tools. The project was conducted in accordance with the PRISMA (Preferred Reporting Items for Systematic Reviews and Meta-Analyses) guidelines [25]. A comprehensive search strategy of subject headings and keywords included “obesity,” “nutrition,” “peer behavior,” and “adolescents” (see Multimedia Appendix 1 for full set of terms).

### Inclusion and Exclusion Criteria

Inclusion criteria for articles to be included in the review were as follows: study sample included adolescents 10 to 19 years of age; study examined a social media app; study had a cross-sectional, qualitative, observational, and experimental design; study had a social media component; study examined adolescent peer communications in a social media environment; and study examined eating behaviors. Studies were required to be written in English and were published from journal inception to October 2019. Conversely, studies related to the impact of social media on body image, related to gastric bypass, or conducted in animal models were excluded. EndNote X9 (Clarivate Analytics) was used to manage the bibliographic data. All references were downloaded to Google Sheets for screening. Full texts were retrieved and a Google Form was created for data extraction.

### Data Extraction

Two independent researchers (NT and AS) screened the articles, assessed them for eligibility, and extracted the data from the search results. The extracted data were exported to Microsoft Excel 2016 for data analysis. Specifically, extracted data included author names; year of publication; country of study; study time frame; participant ages and genders; total number of participants; total number of user accounts; racial and ethnic groups, including percentage or whole number by group; study design; type of social media; behavior influence on food; and primary study outcomes measured. Reviewer agreement was reached through discussion with the senior author and review of the abstracts. Reviews of the study titles, abstracts, and full text, where needed, were completed to ensure agreement with study inclusion parameters to confirm eligibility. Tiebreakers were decided by DV.

## Results

### Overview

**Figure 1:**
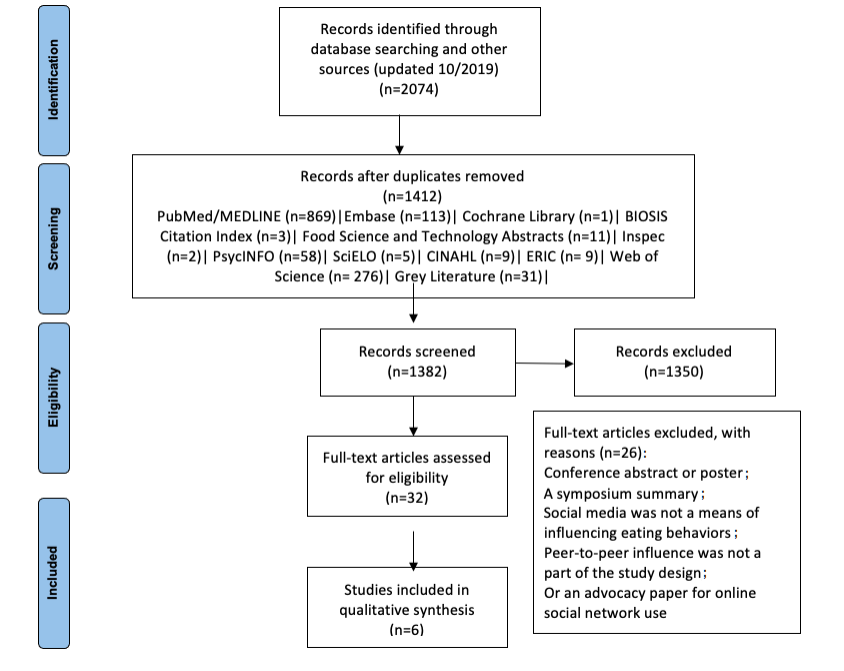
PRISMA (Preferred Reporting Items for Systematic Reviews and Meta-Analyses) flowchart for the articles that met the final inclusion process. Grey literature includes The New York Academy of Medicine Grey Literature Report and the OAIster database from the OCLC (Online Computer Library Center). ERIC: Education Resources Information Center; SciELO: Scientific Electronic Library Online.

Figure 1 shows the PRISMA flowchart for the article selection process for this review.

A total of 1225 adolescents ranging in age from 10 to 19 years who participated in studies across the United States, the United Kingdom, Sweden, Norway, Denmark, Portugal, Brazil, and Australia were represented in the sample. Social media platforms included Facebook and Facebook Messenger, YouTube, Instagram, Twitter, self-made blogs, school websites, and researcher-moderated forums. Each study examined how youth utilized such social media platforms to communicate ideas regarding food and diet.

A total of 2074 articles were identified through electronic databases and manual hand searching of articles from systematic review reference lists. After removing duplicates, 1412 articles remained and were evaluated according to our inclusion criteria. Of the 32 full-text articles that were reviewed further, 26 did not meet the inclusion criteria and were removed. A total of six studies met the full inclusion criteria and were included in the final sample (Figure 1). Table 1 shows the details of these six studies [26-31].

Instagram and Facebook were among the most popular social media platforms that influenced healthful eating behaviors (ie, fruit and vegetable intake) as well as unhealthful eating behaviors related to fast food advertising. Online forums served as accessible channels for eating disorder relapse prevention among youth. However, self-made blogs on anorexia also promoted content about self-harming behavior in support of the eating disorder.

Both positive and negative influences were found in eating behavior content among adolescents. Holmberg et al [26,27] were the only researchers to leverage a contemporary social media platform (ie, Instagram) and identify positive eating behaviors promoted among adolescents. Their study setting across Sweden, Norway, and Denmark is a leading example of the utility of social media to influence eating behaviors in a positive way. Their engagement strategy with adolescents could be leveraged for future studies that evaluate actual eating behavior change. Food safety behaviors were the other positive aspect of healthy eating promoted via social media in the United States, as identified by Quick et al [28]. Kendal et al [29] described self-made blogs for relapse prevention of eating disorders. Fast food advertising, as identified by Thaichon and Quach [30], and self-harming anorexic eating behavior content, as identified by Castro and Osório [31], had negative influences on adolescent eating behaviors (Table 1).

Thematically, results of the six studies included in the scoping review revealed several core themes related to engagement and dissemination of food-related content in a social media environment among adolescent users across eight developed countries. Social media influence converged around four central themes: (1) visual appeal, (2) content dissemination, (3) socialized digital connections, and (4) adolescent *marketer* influencers. Social media not only served as a communication channel to a target cohort but also allowed for interaction through a platform that allowed for user autonomy on a specific topic.

**Table 1:** Data extraction of key variables from each of the six studies that met the final inclusion criteria.

Authors	Age group (years)	Country of study setting	Social media engagement and theme	Main outcomes	Participants
Castro and Osório [31]	13-19	Portugal and Brazil	11 Portuguese-language blogs in Portugal and Brazil Three categories analyzed: Self-harming content Celebrities and fashion models as body image “thinspiration” Proanorexic testimonials about dealing with parental, peer, and other social and cultural offline pressure	The internet is a powerful means of supporting the *proanorexic* movement. This contributes to consumption and production of problematic blog content.	2 boys and 9 girls
Quick et al [28]	11-13	United States	Facebook, YouTube, Oovvuu, and Skype Theme: Food safety promotion videos to improve behaviors among American middle schoolers were disseminated via peer social media networks	Viewing the videos increased perceived susceptibility of food-borne illness and increased self-confidence in performing food safety behaviors.	21 boys and 23 girls
Kendal et al [29]	10-19	United Kingdom	Engagement: 420 message postings 119 usernames; 97 threads Platform: one self-made online forum Themes: Mentorship Online forum as a safe space Friendship within the online forum Flexible help Peer support for recovery and relapse prevention	Online discussion forum was used to help manage eating disorders and overcome maladaptive eating behaviors.	119 unique usernames
Holmberg et al [26]	14	Sweden, Norway, and Denmark	Engagement: 3479 Instagram images 1712 available accounts 1001 Instagram accounts with the hashtag #14 (pertaining to age groups)	Food images were found in most adolescent accounts. Food was often centrally placed and framed in a positive way. Images of food with high calories and low nutrients were framed as advertisements. Images depicting fruits and vegetables were often zoomed in on, similar to images found in cookbooks.	1001 unique Instagram accounts
Thaichon and Quach [30]	11-16	Australia	Platforms: Facebook and Twitter Themes: Peer pressure: users try to match their page through like and share functions via social networks Eating habits and intentions: children said that they tend to change their eating habits after repeatedly being exposed to advertisements on social networking sites	Online marketing via social media sites had a strong impact on children’s decisions to consume fast food.	15 boys and 15 girls
Holmberg et al [27]	13-16	Sweden	Platform: Instagram Engagement: semistructured interviews that described adolescent social media engagement with food Themes: Protecting self-esteem by not disclosing body weight, body images, or unhealthy foods, to minimize the risk of receiving hurtful comments Props and symbols that had positive associations among their peers, which signiﬁed social status, that could generate likes and positive comments were favored	Depictions of body image and food self-presentation in digital social media were the most prominent influential imagery.	11 girls and 9 boys

### Theme 1: Influence of Visually Appealing Imagery and Endorsements

First, communicating eye-catching visual imagery of food, fitness, and body ideals among adolescent peers played a primary role of influence in several modalities. Videos and pictures allow for creative design feature elements that may increase engagement. Entertaining videos with appealing graphics, relatable scenarios, and music were employed effectively in Quick et al’s development of food safety videos. These videos increased food safety practices (ie, handwashing) among 332 preadolescent peer groups in an experimental design study within the United States [28]. Similarly, Holmberg and colleagues [26] reported that shared images of food in social media may reflect a lifestyle that adolescents admire or want to promote. Positive framing of fruits and vegetables that were colorful and aesthetically pleasing may be indicative of a certain status worth sharing [26]. Sharing food images or videos that were perceived as preferable by peers reflect endorsements and may encourage the likelihood of behavior adoption.

Similarly, *likes* or other affirmative visual icons, such as hearts and smiley emojis, connote positive agreement and affirm approval of what is depicted by a statement or image. Food brands with positive associations among peers that signified social status and generated *likes* and positive comments were viewed as favorable among teens in Sweden. In a qualitative study with Swedish teens, one 14-year-old participant described her reasoning for sharing a Starbucks image as follows: “Even if one has never had a Starbucks beverage or visited the place, one still loves it, because one knows that everyone else loves it” [27].

Peer group reinforcers may influence in both directions of the pendulum. Findings of semistructured interviews with 20 Swedish adolescent boys and girls enrolled in a pediatric obesity clinic revealed that they avoided posting unhealthy “fattening” foods in fear of this behavior being viewed as unacceptable from their peers with the potential to elicit criticism and bullying [27]. Posting visual images of healthy foods was used to portray acceptability, as these foods would be viewed favorably and positively by peers. Conversely, Castro and Osório [31] reported that attractive imagery may also influence aspirational ideals toward thinness—or “thinspiration”—among teen bloggers in Portugal and Brazil on proanorexic websites [31].

### Theme 2: Social Media Dissemination

Second, posting images and videos with friends allows for quick dissemination of health-related ideas, products, and practices. Social media has the ability to be circulated worldwide instantly. Rapid dissemination allows for contagion *virality* or *viral marketing* of a topic faster than a formal broadcasting channel, indicating the speed of dissemination by sharing unique, entertaining messages on one’s pre-existing social network [28]. Quick communication uptakes may bypass mainstream media channel dissemination and speed.

The emotion conveyed behind *viral* messages may influence person-to-person well-being. Social media dissemination of harmful ideas may have negative consequences on adolescent mental health, thereby influencing nonpathological eating disorders. Social media contagion passed between individuals may have mental health implications contributing to unique social norms that affect anxiety levels due to *viral* messaging of negative eating behaviors.

### Theme 3: Socializing Digital Connections

Third, digital platforms facilitate peer-to-peer interactions in ways that are quicker and more convenient than traditional in-person support networks, potentially influencing peer social norms. Social media interactions occur at a speed, engagement, and influence level beyond in-person communication. Their reach and scale could effectively influence adolescent beliefs, attitudes, and norms around eating behaviors at a broad scale. Digital communities have been leveraged by the food industry as a marketing tool to advertise directly to consumers. Thaichon and Quach [30] reported that food advertising to young consumers associated a company’s product with community and socialization. The authors found that fast food advertising on social media influenced adolescent views toward fast food, eating habits, and purchasing likelihood [30].

Socialization of digital connections builds online community relationships that connect individuals based on shared experiences, including eating behaviors. Social media digital communities have had positive and negative effects on eating disorder behaviors among adolescents. Castro and Osório [31] were able to engage adolescents in real time across 11 Portuguese-language blogs in Portugal and Brazil in challenges with anorexia through shared cultural pressures and struggles of living with an eating disorder [31]. Similarly, Kendal and colleagues [29] were able to garner peer support in the form of proactive self-care for relapse prevention of anorexic eating behaviors through online forums among adolescents in the United Kingdom [29]. Online access any time of day served as an accessible resource for peer support that allowed for flexible support, friendship in a “safe environment,” and peer support for relapse prevention [29].

### Theme 4: Adolescent Influencer Marketer

Images shared by adolescents with one another may be more influential than commercial advertising. Adolescent user-generated food content on social media was presented and received differently than food advertisements, but still mimicked that of food advertisers. Holmberg et al [26] examined how teenagers presented the food they posted online, analyzing trends in food and drink items and how they were described. The authors found most of the food images (68%) depicted high-calorie, nutrient-poor foods, and only 22% of images included fruits and vegetables. Topic engagement allows for peers to relate to each other through common interest and language. Online relationship building is fostered regardless of differences, such as weight status, which could be rendered absent in a digital world of user self-generated content. In comparison, Thaichon and Quach [30] analyzed how the presence of fast food advertisements on social media influenced the dietary opinions of the adolescents who viewed them. Their exploratory qualitative data found that peer communication on social media was a highly influential factor on purchasing behavior, attitudes toward fast food, and eating behaviors. Table 2 [26-31] shows the influences and eating behavior outcomes related to various social media channels.

Thematic review of the selected articles also revealed a mixed pattern of effect of peer influence on eating behavior of adolescents in social media environments. Social media channels were found to be used as social support for both positive and negative eating patterns. Peer-to-peer support for overweight or obese female adolescents who used Facebook Messenger increased their positive perception about social support and, thus, their online social interactions compared to a group that only received large-group face-to-face support [32]. Positive outcomes were also noted in a moderated online forum for adolescents with eating disorders [29]. The digital modality offered assistance in ways that more traditional services could not, such as by enhancing choice, privacy, and control. Conversely, self-harming proanorexic online content was found within a small group of adolescent blogs in Portugal and Brazil. Peer pressure, need for acceptance, and conflicts with parents were social and cultural pressures that youth were grappling with online [31].

**Table 2:** Influences and eating behavior outcomes by social media channel.

Social media channel	Influences and/or outcomes
Facebook and Twitter	Fast food advertisements can influence young children by the promotion of fast food products and complimentary toys [30].
Instagram	Adolescents presented food images with lifestyle depiction intention in mind to their peers. Positive connotation of fruits and vegetable posts were found. Conclusions were limited due to images not fully representing daily eating. Outcome: food items presented in adolescent social media content and how they were presented were measured [26]. How food items were presented (ie, still-life photos): 20% of food items were arranged as an exhibition, 37.2% were branded food images, and 74.8% included positive adjectives and symbols. What items were presented (ie, types of food): 67.7% of images contained high-calorie, low-nutrient foods and 21.8% contained fruits, vegetables, and berries. Fruit and vegetable images were generally depicted as more visually appealing based on camera zoom and captions. Food items and props were used to protect body image and self-esteem [27].
Various self-made blogs	Reading proanorexia, blogs had no effect on dietary consumption. Content analysis of proanorexia blogs suggests that adolescents pursue harmful minimal food consumption as a result of social and cultural body image, peer pressure and bullying, celebrity and fashion model “thinspiration,” and general exposure to thin-ideal imagery. The blogs themselves contain numerous proanorexia resources and “tips” [31]. This suggests that the internet and social media can serve to promulgate harmful and extreme dietary ideas, although actual behavioral effects were not studied [29].
Online peer-networking eating behavior interventions	Peer-created intervention materials have the potential to reinforce positive nutrition behaviors related to weight loss and food safety among adolescents [28].

## Discussion

### Principal Findings

The literature on peer-enhanced social media interventions for eating behaviors is in its nascent stages. This scoping review aims to fill the gap in the literature and to review the evidence on the influence of peer-to-peer enhanced social media environments on eating behaviors among adolescent youth aged 10 to 19 years. Self-reported, user-generated eating behavior content on social media, supplanted with image recognition, food diaries, nutrient-intake mobile apps, or data synced to wearable devices, such as cameras embedded in eyeglasses, allows for passive data collection with minimal user burden; this data could be integrated into social media in order to build medical evidence to support decision making. Our paper demonstrates that peer social media influence on dietary behaviors warrants a robust amount of additional work to add to the body of scientific medical evidence in the field.

Holmberg and colleagues [26] reported positive portrayals of healthy eating promoted by adolescents. Fruit and vegetable images that are zoomed-in on and focused on for a picture may place emphasis on the food depicted, due to visual appeal and positive attributes. Poelman et al provide an example of a digital food tracking system that could be embedded into social media apps to understand how food choices are influenced by the real-world food environment [33]. Another option is the use of digital food record mobile apps, such as FitNinja (Vibrent Health), with image recognition software to collect nutrient content; these have been found to be acceptable tools for digital food records of real-world food intake [34]. Additionally, shared food posts, such as fruits and vegetables marked by peer *likes* among user networks in social media environments, may represent reinforcement of positive—or any valence—nutrition behaviors as positive, well-liked behaviors [35].

Commercial advertising on Facebook and Twitter, as described by Thaichon and Quach [30], may detract from adolescent engagement, as teens may seek to declare their independence outside of the mainstream; in addition, these platforms are targeted to older age groups. Social media platforms may allow teens a digital environment for creative license, personal identity, and autonomy during a time frame when they are transcending into early adulthood and away from parental influence [7]. In addition, Instagram and Snapchat, which were launched in 2010 and 2011, respectively, are messaging apps whose early adopters are nearly a generation younger than Facebook users. Facebook may not be as relatable, given its inception with a college cohort in 2004, a generation currently approaching middle age.

Peer influence via social media could be an effective channel to engage this typically hard-to-reach population on health topics, including health behaviors. Social media networks were a consistent setting for engaging adolescents with healthy eating messages [36]. Visual appeal was a strong engagement characteristic that influenced users both positively and negatively. Unfortunately, fast food advertising is also pervasive and influential on social media channels targeting adolescents, which could have negative consequences on weight status and other chronic disease risks [37].

Facebook was the most common social media network reported, despite the rising popularity of Instagram and Snapchat over Facebook among adolescents [6]. Only one Swedish study [26] analyzed adolescents’ perception of food on Instagram. This may be due to the time lag in research. One advantage to this could be that as adolescents move away from Facebook, they may be less exposed to the commercial fast food marketing commonly reported on that social media channel.

Healthy eating posts may reflect an aspirational lifestyle change among people in the contemplation phase toward healthy eating. Kinard [38] and Holmberg et al [27] found that obese and overweight adolescents and adults were more likely to engage with healthy food posts than with unhealthy *junk food* posts on Instagram and Facebook [38]. Similarly, Holmberg et al [25] commented that fruits and vegetables were portrayed in a favorable way that connoted palatability. Health promotion marketing of healthy foods may aid to inspire healthful behavior change as users are drawn to the visual appeal.

As social norms are modified in a digital milieu, cautionary monitoring of peer pressures may be needed. Social media peer pressures may affect body image ideals [39] that could lead to maladaptive eating behaviors and poor well-being.

### Healthful Social Media Interventions for Adolescent Eating Behaviors

Multipronged interventions with in-person and social media components have reported successful weight loss among participants [40] and an increase in feelings of social support in adolescent populations [32]. Kulik and colleagues [32] reported that social networking builds peer social support for weight loss in conjunction with an in-person intervention. Peer support may offer teens safe space to share emotional vulnerability, where they can relate to and confide in peers, while also serving as a source of accountability for healthful dietary goals. Similarly, significant weight loss was found as a result of a weight loss intervention that used Facebook private messaging and text messaging among a diverse group of college students [41]. Additionally, Barragan et al found that social media platforms (ie, Facebook, Twitter, and YouTube) increased knowledge on excess calorie intake from sugar-sweetened beverages and increased self-reported intention to reduce sugar-sweetened beverage consumption [16]. Additionally, online discussion forums served as a source of mental health support for eating disorder recovery and relapse prevention [29].

Nutrition information may raise awareness and promote nutrition literacy when content is verified. Mixed messages in the media on the healthfulness of certain foods may be misleading to the public [42]. Additionally, dietary information shared on social media is oftentimes misaligned with national dietary guidelines and evidence-based dietary recommendations. Nutrition content on social media needs to be both accurate and engaging to avoid increasing consumer confusion and skepticism of dietary advice altogether [43]. Public health practitioners, nutrition educators, and researchers need to partner with food industry advertisers, social media influencers, and social marketing leaders to ensure that consumers are accurately informed, particularly for vulnerable populations such as adolescents.

### Negative Effects of Social Media on Adolescent Eating Behaviors

Social media may influence poor eating habits and maladaptive eating behaviors. Thaichon and Quach [30] reported an association between overweight and obese Australian adolescents and behavior intent toward eating fast food due to advertisements viewed on Facebook. Incentive advertising combined with fast food and soda endorsed by their peers may reinforce the promotion of unhealthy food choices. Additionally, two European studies [29,31] engaged adolescents around maladaptive eating behaviors related to eating disorders. Users provided each other with tips and strategies for bulimic or anorexic eating behaviors, promulgating harmful eating behaviors and extreme diets.

### Limitations of the Current Literature

Unfortunately, these studies do not help in understanding the role of social media influence or impact in real-world dietary behavior change in adolescent peer groups. Measurements of actual behavior change need to be studied in conjunction with social media marketing campaigns (eg, purchasing behavior and food intake). Hawkins et al [44] reported that perceived norms and preferences around eating among a sample of English university students (mean age 22 years) on Facebook were predictive of users’ actual food consumption [44]. Facebook users’ perceived social norms were predictive of users’ actual fruit and vegetable intake, and perceived social norms were predictive of participants’ actual snack and sugar-sweetened beverage consumption. Also, MySpace and Reddit were not included as social media platforms in the search terms list. Omission of MySpace may account for reduced representation by Black and other racial and ethnic minority groups.

### Future Directions

Future research should emphasize methodological rigor to elucidate peer influence on dietary behavior change. An extensive amount of research is needed in the field, including objective measures of actual dietary intake with social media interventions and social network analysis of peer influence change agents on food behavior outcomes. In a pilot study that examined whether promoting red peppers via a social media influencer on Instagram would increase actual vegetable intake among adolescents in the Netherlands, no effect was found on users’ actual dietary intake [45]. Additional work is needed to understand the influence of peer-to-peer behavior transmission and adoption in social media environments. The lack of appropriate medical evidence to support decision making might be resolved with more research studies utilizing social media channels alongside objective eating behavior measures. Social media geographic location check-in tools could build off of this approach.

Additionally, fact checking of user-generated content and use of credible dietary sources on social media may be questionable. Content verification of nutrition information [18] may also be affected by perception of friendship ties [46]. Perceived degrees of connection and measurement, or lack thereof, of health outcomes are also limitations when understanding the utility of social media use for adolescent health behaviors [46].

Future research may also include Snapchat and other novel platforms that are now pervasively used by youth [6]. TikTok is also a popular social media platform that was released in 2016 by ByteDance.com that is gaining popularity, particularly during the COVID-19 global pandemic. This video sharing social networking service started in China and gained traction in the United States in 2018 after merging with musical.ly. The social media channel allows users to create short lip sync, dance, and comedic videos [47].

Racial and ethnic youth of color are underrepresented in studies of this kind. Only Kulik et al, who conducted a study in the United States, included minority youth; in their study of Facebook as a complement to an in-person weight loss intervention, 20% of the sample was African American and 21% were participants from other groups of color [32]. Since non-Hispanic Black (22%) and Hispanic (26%) youth experience obesity rates consistently higher than their White counterparts (14%) [48], more research is needed to understand the impact of social media influence on eating behaviors in adolescents of color.

Racial and ethnic health disparities experienced by people of color give rise to a heightened need for targeted healthful marketing via social media channels to engage youth. Racial and ethnic minority youth are heavily targeted for fast food marketing [49,50], and communities of color tend to be inundated by food swamps (ie, an abundance of fast food restaurants concentrated in a ZIP Code). Therefore, in order to act against these high-calorie, nutrient-poor advertising messages [51], culturally tailored approaches are needed to promote healthful eating behaviors among this population [52]. In addition, health literacy has been identified as a key social determinant of health among adolescents [53]. Accurate nutrition-related health literacy conveyed through photos, video imagery, and text is critical to addressing diet-related comorbidities among adolescent youths of color.

Future research should evaluate the role of social media engagement with peer influencer change agents in dietary behavior change interventions. The pervasiveness of social media usage among adolescents calls attention to a communication channel that cannot be ignored. Moreover, the cell phone technology that allows touchscreen access to social media may enhance the capacity of peer influencer change agents that could be more powerful than prior print or television media. In the social media realm, evidence from social network analysis indicates that peer influencers are effective health behavior change agents based on leadership styles by peers, social network connectedness, and communication patterns between the peer influencer change agent and end recipient [54]. Even prior to the global popularity of social media, peer influencers were highly regarded change agents. Peer educator change agents were the most commonly used HIV prevention framework, as peer change agents were more likely to be recognized for their leadership qualities [55]. Gender differences may also be explored in future research about adolescent influence on eating behaviors in social media environments. Constant cell phone engagement offers a technology medium that could not only engage adolescents about eating behaviors but could also support adoption of targeted change behaviors.

### Conclusions

Social media offers the potential of a hand-held change agent. Social media use on cell phones has become a global mainstay in contemporary culture, particularly for adolescents. Adolescent youth can serve as digital beacons of influence on health topics, including eating behaviors. Drawing from influencer marketing strategies in the digital landscape, tailored for culture and audience, adolescents could have a significant influence on the health behaviors of their peers. Health promotion initiatives to influence adolescent youth should consider the integration of social media channels.

## Appendix

Multimedia Appendix 1 List of the full set of search terms.

## Abbreviations

ERIC: Education Resources Information Center
MeSH: Medical Subject Headings
OCLC: Online Computer Library Center
PRISMA: Preferred Reporting Items for Systematic Reviews and Meta-Analyses
SciELO: Scientific Electronic Library Online
