# Supplementary material for: Adolescent Peer Influence on Eating Behaviors via Social Media: Scoping Review
Source: J Med Internet Res. 2021 Jun 3;23(6):e19697. doi: 10.2196/19697 (PMC8212626; doi:10.2196/19697)
Supplement: Multimedia Appendix 1 [file jmir_v23i6e19697_app1.docx]

Appendix 1

Search Terms List

“(obesity OR pediatric obesity OR childhood obesity OR adolescent obesity OR body weight OR overweight OR food OR feeding behavior OR eating habits OR eating behavior OR food habits OR diet habits OR dietary habits OR eating OR nutrients OR nutrition OR nutritional status OR child nutrition sciences OR child nutrition science OR adolescent nutrition sciences OR adolescent nutrition science OR calories OR caloric intake OR body image) AND (prevention OR health promotion OR primary prevention OR preventive health services OR health campaigns OR wellness programs OR comparative effectiveness research OR cross cultural comparison) AND (minorities OR ethnic groups OR ethnic group OR minority group OR minority groups OR blacks OR African American OR African americans OR latino OR latinos OR Latinas OR Latina OR latinx OR Hispanic OR Hispanics OR Hispanic Americans OR Hispanic American OR health disparities) AND (social media OR social networks OR internet OR facebook OR instagram OR youtube OR social network OR social networking OR twitter  OR “web 2.0” OR online OR web based OR web media OR instant messaging OR instant messages OR social support OR social capital OR social values) AND (peer OR peers OR peer influence OR peer behavior OR peer group OR peer groups) AND (youth OR teens OR teen OR teenagers OR teenagers OR young adults OR young adult OR adolescent OR adolescents OR adolescence OR child OR children).”
